# The International Rare Diseases Research Consortium: Policies and Guidelines to maximize impact

**DOI:** 10.1038/s41431-017-0008-z

**Published:** 2017-11-20

**Authors:** Hanns Lochmüller, Josep Torrent i Farnell, Yann Le Cam, Anneliene H Jonker, Lilian PL Lau, Gareth Baynam, Petra Kaufmann, Hugh JS Dawkins, Paul Lasko, Christopher P Austin, Kym M Boycott, on behalf of the IRDiRC Consortium Assembly

**Affiliations:** 1 Institute of Genetic Medicine, Newcastle University, Newcastle Upon Tyne, UK; 2 Autonomous University of Barcelona, Barcelona, Spain; 3 EURORDIS-Rare Diseases Europe, Paris, France; 4 IRDiRC Scientific Secretariat, Inserm US-14, Paris, France; 5 Genetic Services of Western Australia, King Edward Memorial Hospital, Perth, WA Australia; 6 Western Australian Register of Developmental Anomalies, WA Perth, Australia; 7 National Center for Advancing Translational Sciences (NCATS), National Institutes of Health, Bethesda, MD USA; 8 Office of Population Health Genomics, Public Health Division, Department of Health, Government of Western Australia, Perth, WA Australia; 9 Department of Biology, McGill University, Montréal, QC Canada; 10 Children’s Hospital of Eastern Ontario Research Institute, Ottawa, ON Canada

## Abstract

The International Rare Diseases Research Consortium (IRDiRC) has agreed on IRDiRC Policies and Guidelines, following extensive deliberations and discussions in 2012 and 2013, as a first step towards improving coordination of research efforts worldwide. The 25 funding members and 3 patient umbrella organizations (as of early 2013) of IRDiRC, a consortium of research funders that focuses on improving diagnosis and therapy for rare disease patients, agreed in Dublin, Ireland in April 2013 on the Policies and Guidelines that emphasize collaboration in rare disease research, the involvement of patients and their representatives in all relevant aspects of research, as well as the sharing of data and resources. The Policies and Guidelines provide guidance on ontologies, diagnostics, biomarkers, patient registries, biobanks, natural history, therapeutics, models, publication, intellectual property, and communication. Most IRDiRC members—currently nearly 50 strong—have since incorporated its policies in their funding calls and some have chosen to exceed the requirements laid out, for instance in relation to data sharing. The IRDiRC Policies and Guidelines are the first, detailed agreement of major public and private funding organizations worldwide to govern rare disease research, and may serve as a template for other areas of international research collaboration. While it is too early to assess their full impact on research productivity and patient benefit, the IRDiRC Policies and Guidelines have already contributed significantly to improving transparency and collaboration in rare disease research.

## Introduction

**Fig. 1:**
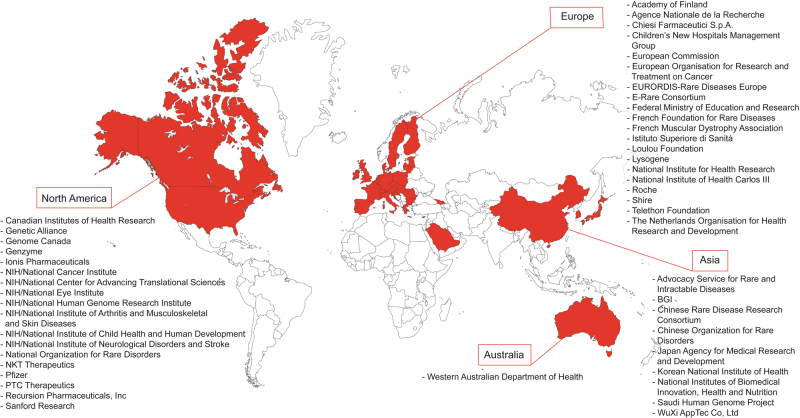
IRDiRC members across the different continents. IRDiRC was launched in 2011 with ~30 members and by mid-2017, counts nearly 50 organizations from Asia, Middle East, Australia, Europe, and North America as members

**Fig. 2:**
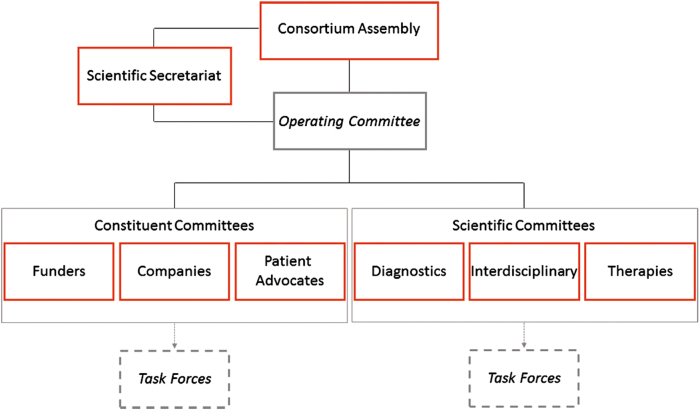
IRDiRC organigram. IRDiRC functions through a Consortium Assembly, an Operating Committee, three Constituent Committees, three Scientific Committees, a number of Task Forces, and a Scientific Secretariat

The International Rare Diseases Research Consortium (IRDiRC) was launched in 2011 to foster international research collaboration and investment in the field of rare diseases, with the aims to contribute to the development of 200 new therapies and the means to diagnose most rare diseases by the year 2020 [1]. IRDiRC is presently a consortium of nearly 50 funding and patient organizations in 20 countries (Fig. 1) that has the ultimate goal to improve diagnosis and therapy for patients affected by rare diseases [2]. Briefly, the composition of members reflected the implementation of the IRDiRC concept from the high level discussions between the United States of America National Institutes of Health (NIH) and the European Commission (EC), recognizing the need to bring together research funders, policy makers, regulators, patient organizations, premier research institutes, and industry to represent almost 350 million people living with a rare disease. IRDiRC currently functions through a Consortium Assembly (formerly Executive Committee), three Constituent Committees (Funders, Companies, Patient Advocates), three Scientific Committees (Diagnostics, Interdisciplinary, Therapies), an Operating Committee, ad hoc Task Forces, and a Scientific Secretariat (Fig. 2) [3].

In early 2012, members of the then IRDiRC Executive Committee, met in Brussels and decided to promote IRDiRC’s mission through a set of policies and guidelines that emphasize collaboration in rare disease research. By early 2013, a draft Policies and Guidelines document, composed of principles based on recommendations from the three IRDiRC Scientific Committees on best practices for rare disease research, was presented to the 25 funding members and 3 patient umbrella organizations. The IRDiRC Policies and Guidelines were discussed point-by-point in April 2013 in Dublin, Ireland, subsequently agreed and adopted, and made available online on the IRDiRC website [4]; the rationale is outlined below and the policies and guidelines are summarized in Table 1. Every member organization joining IRDiRC is required to commit to implementing the Policies and Guidelines through their funding and/or conduct of rare disease research programmes, while respecting existing national legislation and organizational requirements.

**Table 1:** IRDiRC Policies and Guidelines

Type	Description
*Generalized principles*	
Policy	Rare diseases research should be collaborative. Resources, data and results should be shared among IRDiRC research projects and made publicly available to the broader community, and duplication should be avoided.
Policy	Rare diseases research should involve patients and/or their representatives in all relevant aspects of the research.
Policy	International, national, regional and local legislation/regulations need to be adhered to with respect to data protection and ethical approvals.
Guideline	The impact of research on people living with a rare disease should be a key consideration for each project. Best ethical practices for ensuring the interest of the individuals living with rare diseases should be applied.
Guideline	Information about IRDiRC and associated research projects should be disseminated and made available to the rare diseases communities and the public.
Guideline	Education, training and awareness of stakeholders should be encouraged by IRDiRC.
*Data sharing and standards*	
Policy	Research projects should adhere to standards endorsed by IRDiRC.
Policy	Data producers acknowledge their responsibilities to release data rapidly and to publish initial analyses in a timely manner. IRDiRC members will encourage and facilitate rapid data release.
Guideline	Data generated from research projects, including source data, should be deposited in appropriate open or controlled access public databases.
*Ontologies*	
Policy	IRDiRC members will promote the harmonization, interoperability and open access of ontologies to be applied to databases, registries, and biobanks.
Guideline	Ontologies utilized by rare diseases research projects should build upon existing best practice and allow integration and interoperability across different ontologies, including those for model organisms. Ontologies should include rare disease classification ontology (nosology), a phenotype ontology with comprehensive coverage of rare disease manifestations including laboratory values and imaging, as well as ontologies to support biobanking, clinical trials, and research.
*Diagnostics*	
Policy	IRDiRC members should promote the discovery of all the genes that underlie rare diseases and facilitate the development of diagnostic testing for most rare diseases.
Policy	Research projects should contribute to the development and evolution of standards for rare diseases diagnostic testing and reporting.
Guideline	Research projects should cooperate with efforts to produce a well-curated and interoperable inventory of rare diseases.
*Biomarkers*	
Policy	Research projects should establish criteria and standards for evaluation, qualification and validation of biomarkers.
Guideline	The use of biomarkers in rare diseases therapeutic development should be discussed and agreed with regulatory authorities through established procedures.
*Patient registries*	
Policy	Rare disease patient registries should aim to be global in geographic scope and practice. Interoperability and harmonization between rare disease patient registries should be consistently pursued. Linking and data transfer into existing platforms should be considered “best practice”. Registries should be broad and not focused exclusively around a single therapeutic intervention or product.
Guideline	Rare disease patient registries should be linked with data and biological specimens in biobanks, natural history studies and clinical trials and should include measures of quality control and updating.
Guideline	Patients and/or their representatives should be involved in the governance of rare disease registries.
*Biobanks*	
Policy	Rare disease biobanks should aim to be global in geographic scope and practice. Interoperability and harmonization between rare diseases biobanks should be consistently pursued. Linking and data transfer into existing platforms should be considered “best practice”. Sharing and distributing of biomaterials amongrare diseases biobanks is highly encouraged.
Guideline	Rare diseases biobanks are essential resources and should be sustainable. Rare diseases research studies should utilize biobanks for processing and storage of biomaterials and should include methods of quality control and updating.
Guideline	Patients and/or their representatives should be involved in the governance of rare diseases biobanks
*Natural history*	
Policy	Research projects should contribute to the development and evolution of a set of standards for rare diseases natural history studies. The outcomes of natural history studies should be considered in the design of clinical research.
Guideline	Patients and/or their representatives should be involved in defining the objectives, the design, the outreach, and the analysis of clinical research and natural history studies.
*Therapeutics*	
Policy	IRDiRC members will encourage the development of therapies that could be approved by 2020, while respecting each funding entity’s strategic research agenda (including products with an existing orphan designation, the repurposing of already marketed drugs, or funding preclinical orphan development intended to substantiate proof-of-concept).
Guideline	Clinical investigations supported by IRDiRC funders should meet requirements set by regulatory agencies.
Guideline	Adequate scientific and regulatory information about clinical research should be exchanged by researchers.
Guideline	IRDiRC members should promote collaborative multinational studies, with common study procedures and harmonized policies for regulatory and ethical requirements.
*Models*	
Policy	IRDiRC members should promote coordination between human and model systems research in RD.
Guideline	Prior to proceeding to clinical trials, experimentation providing multiple lines of evidence should be robust, reproducible and sufficiently powered.
*Publication and intellectual property*	
Policy	Research projects should publish their results in a timely manner in peer-reviewed scientific journals, preferably with open access.
Guideline	Research publications should appropriately acknowledge research funding and the use of infrastructures such as biobanks and registries, as well as the contribution of patients and their representatives.
Guideline	IP issues and confidentiality agreements need to be balanced with the need to share information for the benefit of research and the patient community.
Guideline	Rare diseases research should be published even where its outcomes are negative or do not show convincing results, including clinical trials.
*Communication on IRDiRC*	
Policy	IRDiRC members will disseminate relevant information on their research project portfolio through adequate and timely measures, in particular the IRDiRC website.
Guideline	IRDiRC shall publish its mission statement, list of member organizations and list of associated projects. IRDiRC shall publish non-confidential proceedings, as well as the minutes and approved documents of its Executive Committee, the Scientific Committees and the Working Groups.
Guideline	IRDiRC-associated projects and IRDiRC member organizations should make reference to IRDiRC, where appropriate, on organizational websites, information material and presentations.
Guideline	IRDiRC will promote active exchanges, events and activities between stakeholders, including patient organizations.

A summary of IRDiRC Policies and Guidelines on generalized principles, data sharing and standards, ontologies, diagnostics, biomarkers, patient registries, biobanks, natural history, therapeutics, models, publication and intellectual property, and communication on IRDiRC.

### Rationale of IRDiRC Policies and Guidelines

#### Generalized principles

The challenge: much rare disease research has been fragmented and compartmentalized, leading to lack of integration, duplication of efforts, lack of critical mass, thinking in “silos” and inefficient use of resources, therefore hindering progress towards better diagnosis and therapy for rare disease patients. Current regulatory and ethical systems can also be a barrier to collaboration, further increasing the disadvantage to and vulnerability of rare disease patients. Most rare diseases are genetic and chronic, leading to long-term disabilities. They can be difficult to diagnose, most often lack an effective treatment, and usually require specialist care and access to expert centers [5]. Additionally, there are particular difficulties in clinical trials for rare diseases, which involve small patient numbers and frequently lack well-defined outcomes [6]. However, while rare diseases are seen as very diverse and different from one another, commonalities between different rare diseases exist that can be utilized and exploited, and this is best achieved through collaborative approaches, e.g., co-development of model systems common to many rare diseases and sharing that knowledge and expertise. There is an urgent need for better integration of rare disease research, in particular with a view to sharing approaches, resources and data that will enhance the development of better diagnoses and therapies, and not reinvent the wheel [7]. This integration mandates a cultural change while respecting data protection and ethical approvals, and the direct involvement of all relevant stakeholders (scientists, doctors, patients, industry, regulators) to collectively focus on the key outcome which is improved health, through better diagnoses and therapies, for people living with rare diseases worldwide.

#### Data sharing and standards

To achieve the IRDiRC goals of contributing towards the development of 200 new therapies and means to diagnose most rare diseases by 2020, many types of resources and research data will be generated and shared; this will facilitate discovery of genes and treatments while ensuring efficient resource utilization. Ultimately, it is critical to the overall success of IRDiRC that datasets obtained from one project will be directly comparable to datasets obtained from another, even if generated using a different approach or technology [8, 9]. This principle has been further developed into the “International Charter of principles for sharing bio-specimens and data” [10], which is endorsed by IRDiRC as one of the “IRDiRC Recognized Resources” [11]. Other resources and infrastructures for rare diseases research, including DECIPHER, RD-Connect, and PhenomeCentral, have embraced these principles and provide mechanisms for data sharing. Further “IRDiRC Recognized Resources” include the HGVS Nomenclature, which sets forth recommendations to facilitate the report and exchange of genome information; and the guideline “Framework for Responsible Sharing of Genomic and Health-Related Data” [12]. IRDiRC also supports the FAIR Data Principles, that is, data produced and published by researchers should be findable, accessible, interoperable, and reusable [13].

#### Ontologies

Ontologies are a machine readable representation of a domain of knowledge based upon a controlled, standardized vocabulary for describing entities and the semantic relationships between them [14, 15]. Ontologies are increasingly used in different fields in science and medicine as they are extremely valuable for data integration, organization, searching, and analysis. Two of the most important kinds of ontologies for rare disease clinical medicine and research are ontologies of phenotypic features (signs, symptoms, and findings of diseases) [15], and ontologies of diseases and disease groups (nosologies) [16]. It is important that ontologies are interoperable; this is best achieved if there is minimal overlap in the concepts covered by the ontologies (orthogonality) and if the ontologies are semantically compatible with one another [17, 18]. To date, IRDiRC has promoted the use of a number of ontology tools, including Human Phenotype Ontology (HPO) [18], Orphanet Rare Disease Ontology (ORDO) [19], and the set of phenotypic terms provided by the International Consortium of Human Phenotype Terminologies [20].

#### Diagnostics

An accurate molecular diagnosis is essential for informed patient management and family counseling, as well as for rare disease research including natural history studies, biomarker identification and clinical trials. There are ~7000 rare diseases and the relevant gene is known (as of 2016) for approximately half of these, thus around 3500 are still without a defined molecular pathogenesis. In addition, a significant fraction of rare disease patients are without a molecular diagnosis due to a lack of universal accessibility of diagnostic testing. For diagnostic testing to be available for the majority of rare diseases by the year 2020, IRDiRC must focus on the discovery of the genes for the 3500 phenotypes that are currently without an associated disease gene [21]. Another challenge faced is diagnostics beyond the exome, and approaches to overcome these barriers to gene discovery are limited; the development of innovative approaches for discovery is required to solve these unsolved conditions, and IRDiRC aims to gather key researchers and review strategies to address this challenge [21]. International efforts to establish guidelines for the clinical reporting of genomic sequencing in a clinical setting, including the approach to report incidental findings, will expedite the delivery of high-throughput and cost-effective testing to the rare diseases patient community as a whole, e.g., guidelines for diagnostic next-generation sequencing developed by EuroGentest and the European Society of Human Genetics [22]. In addition, the necessary bi-directional flow between the clinic and research will be enabled by the IRDiRC Policies and Guidelines [23].

#### Biomarkers

A biomarker is a measurable biological characteristic that is an indicator of normal biological and pathogenic processes and/or response to therapeutic or other interventions [24]. It has been suggested that the use of appropriate biomarkers can reduce the overall cost of developing new innovative treatments including therapies for rare diseases. Moreover, biomarkers may also enhance the efficacy and safety of new treatments and provide a more rational pathway to facilitate advances in rare diseases preclinical and clinical therapeutic development. However, it should be emphasized that the use of biomarkers in biomedical research has several limitations as they may or may not be correlated with clinical outcomes. The work needed to understand the relationship of biomarker changes to either a clinical outcome or other aspects of a biological system is often substantial and early dialog with regulatory authorities is essential to facilitate successful biomarker translation [25].

#### Patient registries

Patient registries are organized databases where patient information, including demographic, medical, and family history, is collected, stored, and available for retrieval via standardized and secure methods. Patient registries are increasingly recognized as crucial tools for rare disease research [26], but there remains a clear need for their standardization, coordination, and further development. High-quality and standardized clinical data are critical for patient registries which requires the systematic use of ontologies such as the HPO (see above). In particular, patient registries must overcome the following challenges to develop their full potential in rare disease research: (a) lack of harmonization due to high variability among registries according to rare diseases coding systems, geographical coverage, and type of data collected; (b) lack of data sharing since only a minority share data with other databases, biobanks or centers of expertise; (c) lack of sustainability since rare disease patient registries often expire due to lack of commitment from data providers, lack of funding, or study termination, leading to loss of data and loss of investment; and (d) lack of utility for research owing to absence of quality control, standardized data elements, and genetic data.

#### Biobanks

Biobanks are collections of biomaterials with associated data. Biobanking is an essential tool to provide access to high-quality human biomaterial and data for fundamental and translational research. Rare disease research benefits from the provision of human biomaterials through biobanks, and each sample from a person with a rare disease has a high value as it may hold the key to answering an important research question. The rarity and diversity of rare diseases and their associated biomaterials present specific challenges and opportunities for biobanking, requiring transnational collaboration and harmonization [27, 28]. Providing and managing information and access to valuable biological samples through a simple and reliable process is crucial for rare disease research and will contribute to the development of new diagnostic techniques, biomarker development, identification of potential therapeutic targets and testing therapeutic response. Biobanks need to overcome the following challenges to develop their full potential in rare disease research: (a) lack of policy for data sharing, access, attribution and protection, and IT harmonization; (b) lack of biomaterial and data sharing; (c) lack of sustainability; and (d) lack of utility for research.

#### Natural history

Performing natural history studies will facilitate the identification of disease characteristics that can be used when planning and conducting clinical investigations for rare disease therapies. Moreover, this knowledge will also serve to define a trial’s target population, develop biomarkers for disease progression and therapeutic response, determine appropriate surrogate and relevant clinical endpoints, and decide on the study duration. As rare diseases are highly diverse in nature, there is no one set of data elements that can be recommended for data collection in all natural history rare disease studies; rather the disease characteristics should reflect the prominent features of the rare disease [29].

#### Therapeutics

Clinical trials represent a major challenge for the development of therapies intended to treat, cure, or prevent rare diseases. It is well recognized that clinical investigation in this field is associated with several hurdles that may jeopardize the performance of these investigations, when compared with common diseases. Small patient populations, together with geographical dispersion, add complexity to the design and performance of trials aimed at providing efficacy and safety information supporting a marketing authorization/approval of these therapies [30]. However, introduction of rare diseases legislation and orphan designation procedures have brought a large number of investigational products into the development pipeline [31]. Combined efforts are required of investigators, industry, patients’ representatives, research institutions, and regulatory authorities to overcome all bottlenecks associated with clinical trials in low-prevalence conditions. Among the incentives provided by orphan designations, protocol assistance and scientific advice from regulatory bodies play a key role in guiding the conduct of studies to address the benefit/risk analysis for marketing authorization and approval [32, 33]. Thanks to these strategies, in the US, 47% and 41% of novel drugs approved by the Food and Drug Administration (FDA) in 2015 and 2016, respectively, were for rare indications [34, 35]. The trend observed in the success rates of clinical development between 2006 and 2015 in the US shows higher rates of approval for orphan products moving on from one phase of clinical studies to another when compared to drugs for chronic conditions [36].

#### Models

Currently, we only understand the biological function of a fraction of human genes. Animal and cellular models, including humanized models, provide insight into the function of genes and the mechanisms underlying rare diseases can be manipulated experimentally much more readily than humans for both ethical and technical reasons, allowing important questions that cannot be addressed in patients to be approached [37]. Model systems enable experimental interventions that can establish causal mechanisms of gene action, thereby putting disease genes into biological context and can yield pathologic insight that facilitates the development of targeted therapeutics.

#### Publication and intellectual property

Rare disease research results should be rapidly shared and made highly visible to the scientific, health care, patient, pharmaceutical, and medical device communities. Their utility must be clearly demonstrated and potential users must have the opportunity to receive training in the techniques and tools developed. This includes negative results, which can be as important for the rare disease field as are new scientific breakthroughs (in relation to data sharing policies and guidelines). Intellectual Property (IP) is an important factor for the public and the private sector, in particular to cover the significant cost of developing new therapies. Issues related to IP rights need to be assessed and handled in accordance with fundamental ethical rules and principles. Tools to handle IP issues may include exploitation and technological implementation plans, non-exclusive licensing, patenting, knowledge property rights, and pre-existing know-how. In many instances, confidentiality agreements may be required between the parties involved.

#### Communication on IRDiRC

New knowledge, tools, and resources are generated through the rare disease research funded or supported by IRDiRC members. Its outputs require high visibility to a range of stakeholders and a clear strategy to train and educate the next generation of scientists and other users. The goals of an external dissemination strategy are to promote international academic and industrial cross-fertilization, both within and outside IRDiRC, and to provide information on IRDiRC research to other research projects, the scientific community, industrial groups, government bodies, policy makers, and the general public, including patients. IRDiRC communication will be built on the principles of openness, public accessibility, transparency, inclusivity, and timeliness. Non-confidential discussions and outcomes of IRDiRC meetings will be reported and published on its website for access by all stakeholders of rare diseases research and not only to participating members, and workshop documents and recommendations will be made available for public consultations.

### Implementation of IRDiRC policies and guidelines

By mid-2017, nearly 50 organizations, including patient advocacy organizations, from 20 countries have become an IRDiRC member and accepted IRDiRC Policies and Guidelines as principles to be implemented in their rare disease research programmes and contribute to IRDiRC’s goals. Some of IRDiRC’s principles have been further developed into more detailed guidance documents, such as the “International Charter of principles for sharing bio-specimens and data” [10], that has been adopted for rare disease research and beyond [38]. Policies related to the development of therapies have led funders to mandate Orphan Drug Designation and Protocol Assistance by regulatory agencies for some of their programmes ear-marked to support clinical trials in rare diseases. More recently, IRDiRC has developed a number of new initiatives, e.g., the “IRDiRC Recognized Resources” program and Scientific Committee-directed Task Forces, to further the impact of the Policies and Guidelines. “IRDiRC Recognized Resources” highlights publicly available resources that are peer-reviewed and have been assessed to comply with the Policies and Guidelines [11]. IRDiRC Task Forces address particular topics or questions related to rare disease research, identify challenges to efficiency and effectiveness in global rare disease research, suggest and/or create solutions including standards and tools, and publish recommendations to further reinforce these policies and guidelines [39]. IRDiRC Policies and Guidelines may also have an impact on member organizations beyond their rare disease programmes, on other initiatives and collaborations, e.g., health system policy implementing HPO, ORPHA nomenclature, and data sharing policies. IRDiRC is investigating additional ways to promulgate the policies and guidelines and assess their impact, and will update them as required.

More broadly from a genomics policy perspective, IRDiRC partners with the Global Alliance for Genomics and Health (GA4GH) to collaboratively develop policy and guidelines around consent and data sharing [40], and frameworks for ethical and secure data sharing [41]. Similarly, IRDiRC is linked with the Global Genomic Medicine Collaborative (G2MC), an initiative to capture and disseminate best practices for genomic medicine in bioinformatics, education, evidence, pharmacogenomics, and policy across the global genomic medicine community. Other partnerships and initiatives specifically targeting rare diseases that are linked with IRDiRC include, but are not limited to: RD-Connect; TREAT-NMD; CARE for RARE Canada; Human Variome Project; RARE-Bestpractices; RD-Action; European Reference Networks (ERNs); the USA Office of Rare Diseases Research with its Rare Diseases Clinical Research Network (RDCRN); and the Undiagnosed Diseases Network International (UDNI). IRDiRC is committed to working broadly with its partners to facilitate delivery of its goals. (See Supplementary document 1 for description of IRDiRC’s partner initiatives).

The role of patient organizations must also be emphasized. Representatives from patient organizations are participants in IRDiRC Committees and Task Forces in order to ensure patients’ views are taken into consideration during strategic planning and on all activities, carried out in line with the agreed principles described in the IRDiRC Policies and Guidelines. Patient organizations that are also funders of research reinforce the implementation of these principles through their funding programmes, external representatives are systematically invited to provide their input on various aspects including recommendations to improve rare disease research policies and practices, and a newly formed Patient Advocates Constituent Committee will identify further common barriers to be addressed through collaborative actions that apply the IRDiRC Policies and Guidelines.

## Conclusions

IRDiRC is focused on accelerating progress in the field of rare disease research through international cooperation and collaboration, with the ultimate goals of enabling the means to provide a diagnosis for all rare diseases patients, and to contribute to the development of new therapies for rare diseases. In order to increase the joint impact of rare diseases investment by funding agencies, industry, researchers, regulators, and rare diseases patient advocates, harmonization of efforts that address common roadblocks is needed. To assist in this task, IRDiRC developed a set of Policies and Guidelines, which are the principles that IRDiRC members agree to adhere to, focused on data sharing and standards, ontologies, diagnostics, biomarkers, patient registries, biobanks, natural history, therapeutics, models, publication and intellectual property, and communications about the Consortium. The IRDiRC Policies and Guidelines are the detailed and worldwide agreements of major public and private funding organizations to govern rare disease research, with the Consortium representing over 2 billion US dollar of investments. While it is too early to fully gauge the depth and magnitude of impact on rare disease research and patient benefit, the IRDiRC Policies and Guidelines have already significantly contributed in improving transparency and collaboration in this field. IRDiRC is now making steps towards addressing gaps and barriers in rare disease research; Task Forces are established to specifically address some of these gaps through policy recommendations and/or technical solutions. Rare disease research has made considerable progress in the last decade, and the IRDiRC Policies and Guidelines will further push the discovery progress of rare disease diagnosis and treatment, thereby advancing this important field of research.

## Electronic supplementary material

Partner Initiatives
