# Supplementary material for: The International Rare Diseases Research Consortium: Policies and Guidelines to maximize impact
Source: Eur J Hum Genet. 2017 Nov 20;25(12):1293–302. doi: 10.1038/s41431-017-0008-z (PMC5865169; doi:10.1038/s41431-017-0008-z)
Supplement: Supplementary file 1 — Partner Initiatives [file 41431_2017_8_MOESM1_ESM.docx]

**Supplementary document 1: IRDiRC’s Partner Initiatives (in alphabetical order)**

**CARE for RARE** is a Canadian research network, funded by the Genome Canada and the Canadian Institutes of Health Research, to enhance care of patients with rare genetic diseases through activities focused on novel gene discovery, clinical translation of genome-wide sequencing, and generalizable approaches to repurposing clinically-approved medications.^1^ [www.care4rare.ca](http://www.care4rare.ca)

**European Reference Networks (ERNs)** for rare diseases are being developed to serve as research and knowledge centres, and aim to provide patients with accurate diagnosis and advice on the best treatment for their conditions.^2^ These virtual networks of European healthcare providers use dedicated platform and tools to review a patient’s diagnosis and treatment, and will also facilitate expertise and knowledge sharing, enable research collaborations and trainings, help accelerate therapy development, and promote development of guidelines and best practices. <http://ec.europa.eu/health/ern/>

**Global Alliance for Genomics and Health (GA4GH)** was established to accelerate progress in genomic medicine and its translation for human health. Over 400 leading institutions in healthcare, research, disease advocacy, life science, and information technology work collaboratively under the following founding principles: respect, transparency, accountability, inclusivity, collaboration, innovation, agility and independence. These principles are reflected in GA4GH’s activities, including the adoption of federated data ecosystem to share genomic and clinical data.^3^ <http://genomicsandhealth.org/>

The **Global Genomic Medicine Collaborative (G2MC)** is an action collaborative that aims to identify opportunities and foster collaborations in the implementation of genomic medicine in clinical care.^4^ Among its activities are the development of genomic medicine projects, the creation of a registry/catalogue of genomic medicine projects to facilitate collaborations, the role as a global genomic medicine policy forum, and the eradication of preventable Stevens Johnson Syndrome. <http://www.nationalacademies.org/hmd/Activities/Research/GenomicBasedResearch/Innovation-Collaboratives/Global_Genomic_Medicine_Collaborative.aspx>

The **Human Phenotype Ontology (HPO)** provides a comprehensive, standardized and structured vocabulary of human phenotypic abnormalities.^5^ It is interoperable with multiple resources, in particular phenotypic databases of model organisms, and includes various meta-attributes including frequency and references. <http://human-phenotype-ontology.github.io>

The **Human Variome Project (HVP)** is an international non-governmental consortium with a central coordination function: that all information on genetic variation and its effect on human health can be collected, curated, interpreted and shared freely and openly.^6^ The HVP has an Associate NGO status with the UNESCO and a Memorandum of Understanding with the World Health Organisation. [www.humanvariomeproject.org](http://www.humanvariomeproject.org)

**ORPHA number** is a unique and stable identifier attributed to each rare disease listed in the Orphanet database to aid disease identification and improving rare diseases visibility in healthcare and research information systems. The ORPHA nomenclature, updated monthly, is also available on Orphadata as an XML file.^7^ <http://www.orpha.net/consor/cgi-bin/Disease_Search_List.php>

**RARE-Bestpractices** is a global platform, funded by the European Commission, to improve the management of rare disease patients through dissemination of clinical practice guidelines, identification of research needs and gaps in rare diseases, and training courses to improve guidelines knowledge and expertise sharing.^8^ <http://www.rarebestpractices.eu/>

The **Rare Diseases Clinical Research Network (RDCRN)** was established in 2003 by the NIH Office of Rare Diseases Research (ORDR), now overseen by the National Center for Advancing Translational Sciences (NCATS), to advance medical rare diseases research by supporting multi-disciplinary teams and patient advocacy groups in clinical studies, and facilitating collaboration, study enrolment and data sharing of over 200 rare diseases across the USA.^9^ <https://www.rarediseasesnetwork.org/>

**RD-Action** is a European Commission Joint Action with the objectives to improve knowledge on rare diseases and orphan drugs, contribute to disease codification for use in European healthcare systems, and to support the development of national and European policies in the field in accordance to priority actions identified by the European Commission Expert Group on Rare Diseases (CEGRD).^10^ <http://www.rd-action.eu/>

**RD-Connect** is a unique, integrated infrastructure, funded by the European Commission, that brings together databases, registries, biobanks and clinical bioinformatics data used in rare disease research into a central and comprehensive resource for researchers worldwide.^11^ It promotes interoperability of resources and infrastructures, develops bioinformatic tools, and provides controlled data sharing and sample access. <http://rd-connect.eu/>

**TREAT-NMD** is a European neuromuscular network that provides an infrastructure to ensure that promising, cutting edge therapies reach patients as quickly as possible.^12^ Launched in 2007, the network focuses on developing tools to translate novel therapeutic approaches from bench into the clinic, facilitating strong international research collaborations, and establishing guidelines and best practices in the care of neuromuscular patients worldwide. <http://www.treat-nmd.eu/>

The **Undiagnosed Diseases Network International** (UDNI) has built a consensus framework of principles, protocols, best practices and governance to address the needs of undiagnosed patients and gain insights of disease aethiopathogenesis.^13^ International clinical experts from UDNI participating centres employ available resources and know-how to fill the knowledge gaps in order to provide a diagnosis. <http://www.udninternational.org/>

**Supplementary References**

1. Care4Rare Canada, 2017. Available at <http://care4rare.ca/> accessed on 12 April 2017.
2. European Reference Networks, 2017. Available at <http://ec.europa.eu/health/ern/> accessed on 12 April 2017.
3. Global Alliance for Genomics and Health. A federated ecosystem for sharing genomic, clinical data. *Science* 2016;**352**:1278-80.
4. The National Academies of Sciences, Engineering, and Medicine; Health and Medicine Division (HMD). Global Genomic Medicine Collaborative (G2MC), 2017. Available at <http://www.nationalacademies.org/hmd/Activities/Research/GenomicBasedResearch/Innovation-Collaboratives/Global_Genomic_Medicine_Collaborative.aspx> accessed on 12 April 2017.
5. Köhler S, Vasilevsky N, Engelstad M *et al*. The Human Phenotype Ontology in 2017. *Nucl. Acids Res* 2016;**45**:D865-D876.
6. Burn J, Watson M. The Human Variome Project. *Hum Mutat*. 2016;**37**:505-7.
7. Orphadata: Free access data from Orphanet, 1997. Available at <http://www.orphadata.org> accessed on 12 April 2017.
8. Taruscio D, Morciano C, Laricchiuta P, the RARE-Bestpractices consortium. RARE-Bestpractices: a platform for sharing best practices for the management of rare diseases. *Rare Dis Orphan Drugs* 2014;**1**:5-8.
9. Krischer JP, Gopal‐Srivastava R, Groft SC, Eckstein DJ. The Rare Diseases Clinical Research Network's Organization and Approach to Observational Research and Health Outcomes Research. *J Gen Intern Med* 2014;**29**:739‐744.
10. RD-Action, 2016. Available at <http://www.rd-action.eu> accessed on 12 April 2017.
11. Thompson R, Johnston L, Taruscio D *et al*. RD-Connect: An Integrated Platform Connecting Databases, Registries, Biobanks and Clinical Bioinformatics for Rare Disease Research. *J Gen Intern Med* 2014;**29**:S780-7.
12. Bushby K, Lynn S, Straub T, TREAT-NMD Network. Collaborating to bring new therapies to the patient--the TREAT-NMD model. *Acta Myol* 2009;**28**:12-5.
13. Taruscio D, Groft SC, Cederroth H *et al*. Undiagnosed Diseases Network International (UDNI): White paper for global actions to meet patient needs. *Mol Genet Metab* 2015;**116**:223-5.
